# Plastid retrograde signaling: A developmental perspective

**DOI:** 10.1093/plcell/koae094

**Published:** 2024-03-28

**Authors:** Naresh Loudya, Alice Barkan, Enrique López-Juez

**Affiliations:** Department of Microbiology and Cell Biology, Indian Institute of Science, Bengaluru 560012, India; Institute of Molecular Biology, University of Oregon, Eugene, OR 97403, USA; Department of Biological Sciences, Royal Holloway University of London, Egham TW20 0EX, UK

## Abstract

Chloroplast activities influence nuclear gene expression, a phenomenon referred to as retrograde signaling. Biogenic retrograde signals have been revealed by changes in nuclear gene expression when chloroplast development is disrupted. Research on biogenic signaling has focused on repression of Photosynthesis-Associated Nuclear Genes (PhANGs), but this is just one component of a syndrome involving altered expression of thousands of genes involved in diverse processes, many of which are upregulated. We discuss evidence for a framework that accounts for most of this syndrome. Disruption of chloroplast biogenesis prevents the production of signals required to progress through discrete steps in the program of photosynthetic differentiation, causing retention of juvenile states. As a result, expression of PhANGs and other genes that act late during photosynthetic differentiation is not initiated, while expression of genes that act early is retained. The extent of juvenility, and thus the transcriptome, reflects the disrupted process: lack of plastid translation blocks development very early, whereas disruption of photosynthesis without compromising plastid translation blocks development at a later stage. We discuss implications of these and other recent observations for the nature of the plastid-derived signals that regulate photosynthetic differentiation and the role of GUN1, an enigmatic protein involved in biogenic signaling.

## Introduction

Chloroplasts emerged from a cyanobacterial endosymbiont over 1 billion years ago. During the evolution of multicellular land plants from single-celled marine chlorophytes, chloroplast functionalities became integrated into the developmental program of the host organism. Undifferentiated meristem cells became the source of all mature cell types, some of which required photosynthetic function and all of which required elements of chloroplast-based central metabolism. Meristem cells possess a small number of “proplastids”—small chloroplast precursors harboring the chloroplast genome but with few ribosomes and very limited internal membrane structures. Proplastids differentiate into chloroplasts or into a range of other “plastid” types in concert with the differentiation of their host cells (Jarvis and López-Juez 2013; Sierra et al. 2023). Thus, mechanisms arose to promote the transformation of proplastids into chloroplasts and to synchronize that process with the broader program of leaf development and photosynthetic differentiation.

## Chloroplast biogenesis involves 3 major stages

In dicots, the development of chloroplasts from proplastids is initiated very early in leaf development, with immature thylakoid networks already apparent in the shoot apical meristem and leaf primordia (Charuvi et al. 2012). A period of rapid cell division in leaf primordia is followed by a wave of cell cycle arrest and cell expansion that proceeds from the tip to the base of the developing leaf, and the transition of immature to mature chloroplasts is concordant with this wave (Andriankaja et al. 2012). All told, the spatial patterning of the proplastid-to-chloroplast transition in typical dicots does not lend itself to physical separation of different stages for biochemical analysis.

In contrast, the proplastid-to-chloroplast transition in the grasses is conveniently displayed in a gradient along the length of the seedling leaf blade. An intercalary meristem at the leaf base comprises proliferating cells harboring proplastids. These give rise to nondividing cells that differentiate as they are pushed toward the leaf tip, such that successive stages in the transition from sink (i.e. heterotrophy) to source (i.e. autotrophy) are represented from leaf base to leaf tip. This feature has been exploited to describe the cell biological, metabolic, and gene expression changes that accompany photosynthetic differentiation. Foundational studies (Leech et al. 1973; Robertson and Laetsch 1974; Dean and Leech 1982; Baumgartner et al. 1989, 1993) revealed the transformation of proplastids to chloroplasts along the length of the leaf, the confinement of plastid DNA replication and division to the leaf base, and 2 phases of plastid transcript accumulation: plastid mRNAs encoding proteins involved in gene expression—subunits of a bacterial-type plastid-encoded RNA polymerase (PEP) and the plastid ribosome—peak in abundance near the leaf base, whereas plastid mRNAs encoding subunits of the photosynthetic apparatus peak in more apical leaf sections. This shift in the plastid transcriptome results from a shift in the dominating plastid RNA polymerase: a plastid-localized phage-type “nuclear-encoded polymerase” (NEP) dominates early in chloroplast development (i.e. at the leaf base) and transcribes plastid genes encoding PEP (Emanuel et al. 2004; Kusumi et al. 2004; Börner et al. 2015). The resulting increase in PEP yields robust transcription of photosynthesis genes in more apical leaf sections (Mullet 1993). These seminal observations culminated in the view that the proplastid-to-chloroplast transition involves 3 successive stages (Mullet 1993): (i) activation of plastid division, DNA replication, and NEP; (ii) buildup of the chloroplast genetic system (PEP, translation machinery); and (iii) production of the photosynthetic apparatus due to increased expression of both chloroplast-encoded and nucleus-encoded subunits.

**Figure 1.**
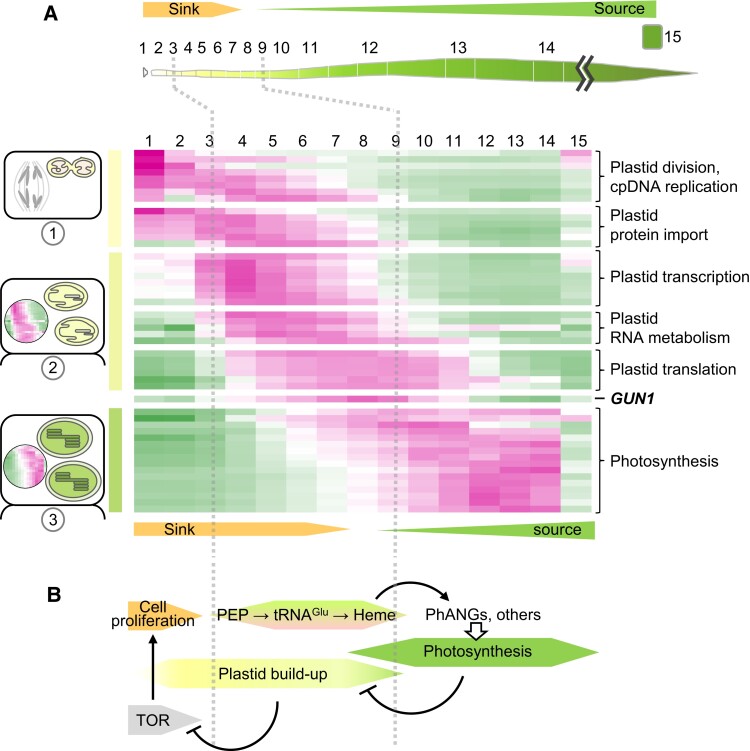
Three phases of chloroplast development as displayed along the length of a monocot seedling leaf, and the effects of retrograde signals on progression through these stages. **A)** The relative abundance (green: low; magenta: high) of mRNAs encoding markers of each phase of chloroplast biogenesis along the developmental gradient of the wheat leaf (Loudya et al. 2021). The developmental dynamics of the orthologous genes in maize (Wang et al. 2014) are similar (Kendrick et al. 2022), albeit less highly resolved. The plastid proliferation phase (1) encompasses plastid division, buildup of the chloroplast import apparatus, and plastid DNA replication. The assembly phase (2) begins with increased expression of genes involved in plastid transcription, followed by buildup of the machineries for chloroplast translation and assembly of the photosynthetic apparatus, as well as initial upregulation of PhANGs. The photosynthesis phase (3) includes peak PhANG expression and declining expression of genes involved in prior phases. The expression profile of *GUN1* is shown to illustrate its timing with respect to the 3 phases. The transcriptomes of maize mutants lacking plastid ribosomes resemble those in wheat leaf Sections 3 and 4 (Kendrick et al. 2022). The data displayed in the heatmap are provided in Supplementary Data Set 1. **B)** A model for 3 biogenic retrograde signaling processes: (1) the upregulation of PEP activity produces tRNA^Glu^, generating the heme signal that promotes expression of PhANGs and many other genes that peak during the photosynthesis phase. (2) The depletion of nucleotides and amino acids by the buildup of plastid ribosomes and by the consequent burst of chloroplast translation inhibits TOR, driving exit from the cell cycle and entry into growth exclusively by cell expansion. (3) The establishment of photosynthesis dampens the expression of genes involved in Phases 1 and 2. See Kendrick et al. (2022) and references herein for the evidence for this scheme. TOR, Target of Rapamycin.

Results from more recent transcriptome, proteome, and ribosome profiling analyses of cereal leaf blades (Li et al. 2010; Majeran et al. 2010; Wang et al. 2014; Chotewutmontri and Barkan 2016; Loudya et al. 2021) have supported and elaborated on this general scheme. Our detailed analysis of the cellular and transcriptome changes along the length of the wheat leaf offered a high resolution in the basal leaf sections, where key developmental transitions are closely spaced (Loudya et al. 2021). These studies support the view that chloroplast biogenesis involves 3 major, albeit overlapping, phases (Fig. 1). An early “plastid proliferation” phase includes plastid division, the majority of plastid DNA replication, and peak expression of genes for NEP and most components of the plastid protein import apparatus. This is followed by an “assembly” phase: buildup of the machineries for plastid gene expression, the targeting of proteins to the thylakoid membrane, enzymes involved in the synthesis of thylakoid lipids and some steps of chlorophyll synthesis, as well as the initial synthesis of plastid-encoded photosynthesis proteins. A subsequent “photosynthesis” phase is characterized by the activation of Photosynthesis-Associated Nuclear Genes (PhANGs; including light-harvesting proteins and CO_2_ fixation enzymes), the peak expression of chloroplast genes involved in photosynthesis, and the establishment of autotrophy. The sink–source transition occurs in conjunction with the transition between the assembly and photosynthesis phases (Li et al. 2010; Majeran et al. 2010; Wang et al. 2014), with over 80% of the chlorophyll accumulating only in the last phase (Loudya et al. 2021). The expression of genes involved in each phase generally declines during the transition to the subsequent phase, resulting in 3 major waves of expression (Fig. 1). Changes in the nuclear transcriptome (Li et al. 2010; Wang et al. 2014; Loudya et al. 2021) are largely congruent with changes in the proteome (Majeran et al. 2010) and are accompanied by changes in the chloroplast translatome (Chotewutmontri and Barkan 2016): the translational output of plastid genes involved in gene expression (PEP, ribosomes) peaks during the assembly phase, whereas the translational output of plastid genes involved in photosynthesis peaks during the photosynthesis phase.

These transitions of the plastid compartment are accompanied by major shifts in extraplastidic properties: for example, cell divisions are confined to the most basal leaf sections where cytosolic translation peaks, the transition to cell expansion coincides with the early chloroplast assembly phase, and investments in mitochondrial functions decline from base to tip (Li et al. 2010; Majeran et al. 2010; Wang et al. 2014; Loudya et al. 2021; Kendrick et al. 2022). Thus, a picture emerges of a regulatory cascade during which the proplastid-to-chloroplast transition plays out in stages in concert with the broader program of leaf development. Based on the limited data available, it seems likely that a similar scenario applies to dicots. For example, examination of the proplastid-to-chloroplast transition during light-induced greening in an Arabidopsis single-cell culture system (Dubreuil et al. 2018) provided evidence for 2 waves of PhANG expression, the second of which follows the activation of PEP.

## Biogenic retrograde signaling

The concept of retrograde signaling was proposed 4 decades ago, following the observation that a barley mutant lacking plastid ribosomes lacks several nucleus-encoded plastid-localized enzymes (Bradbeer et al. 1979). Subsequent work identified additional PhANGs that respond in this manner, established that this involves control at the level of transcription, and showed that antibiotics that inhibit plastid gene expression as well as the bleaching herbicide norflurazon cause similar effects (reviewed in Nott et al. 2006). It was hypothesized that the developmental state of the chloroplast is reported to the nucleus and regulates transcription of PhANGs, serving to coordinate expression of the 2 genomes. Thus the term “retrograde signaling” was born—more specifically, “biogenic retrograde signaling,” to distinguish this phenomenon from “operational signaling,” the communication to the nucleus of stresses perceived by chloroplasts during their function (Pogson et al. 2008).

A breakthrough in understanding the basis of biogenic signaling came from the elegant screen for *genomes-uncoupled* (*gun*) mutants in Arabidopsis: mutants in which inhibitor treatments fail to fully repress PhANGs (Susek et al. 1993). Identification of *gun* loci (Mochizuki et al. 2001; Larkin et al. 2003; Strand et al. 2003) revealed a role for tetrapyrrole biosynthesis in PhANG regulation (reviewed in Larkin 2016; Wu and Bock 2021). This was initially thought to involve the repressive action of an intermediate in chlorophyll biosynthesis, Mg-protoporphyrin IX (Strand et al. 2003), but was later proposed to involve an activating molecule related to heme, whose synthesis increases when the chlorophyll branch of the tetrapyrrole pathway is blocked (Woodson et al. 2011). Over time, evidence accumulated against a repressive role of Mg-protoporphyrin IX and in favor of a heme-related activating signal (Terry and Smith 2013; Woodson et al. 2013; Larkin 2016; Dubreuil et al. 2018; Kendrick et al. 2022; Richter et al. 2023). Although some observations remain puzzling in light of the heme hypothesis, it is emerging as a consensus in the field (see Page et al. 2020; Wu and Bock 2021; Richter et al. 2023). The heme hypothesis provides a straightforward explanation for the influence of chloroplast gene expression on PhANG expression: plastid tRNA^Glu^, the precursor for plant tetrapyrroles (Schön et al. 1986; Kanamaru et al. 2001), is synthesized by PEP (Williams-Carrier et al. 2014), which is synthesized by plastid ribosomes. Therefore, defects in PEP or plastid translation result in loss of heme biosynthesis in plastids, leading to a reduction in PhANG transcription (Woodson et al. 2013). Overaccumulation of chloroplast preproteins in the cytosol has also been proposed as the signal that derepresses PhANGs (Wu et al. 2019), but, as noted previously (Shimizu and Masuda 2021; Loudya et al. 2022), the fact that various mutants with defects in the plastid import machinery express PhANGs at levels similar to that in plastid gene expression mutants harboring wild-type *GUN* loci (i.e. they do not exhibit a *gun* phenotype) makes this possibility less compelling.

In contrast to other *GUN* loci, *GUN1* does not encode an enzyme of the tetrapyrrole pathway (Koussevitzky et al. 2007). GUN1 is a plastid-localized member of the pentatricopeptide repeat (PPR) protein family, whose members typically bind RNA and influence organellar gene expression (reviewed in Barkan and Small 2014). How GUN1 influences biogenic signaling remains a subject of lively debate and is discussed below.

### Beyond PhANGs: retention of juvenile states reprograms the transcriptome

Regulation of PhANG expression has been the focus of most research on biogenic retrograde signaling, but defects in plastid transcription/translation cause many other changes in nuclear gene expression. A long-recognized example is the increased expression of NEP and the plastid genes it transcribes (Han et al. 1993; Hess et al. 1993; Hajdukiewicz et al. 1997; Emanuel et al. 2004; Tadini et al. 2020a). Thus, plants that fail to express PEP (due, e.g., to the absence of plastid ribosomes) exhibit a stereotypical change in plastid transcript populations: the decreased abundance of RNAs synthesized primarily by PEP (most of which are involved in photosynthesis) and the increased abundance of those synthesized primarily by NEP (mostly involved in house-keeping functions). This transcript pattern is sometimes referred to as the *Δrpo* syndrome, as it is observed when plastid *rpo* genes encoding PEP subunits are deleted (Hajdukiewicz et al. 1997).

Numerous transcriptome studies have now shown that reduced PhANG expression and increased NEP expression represent just the tip of the iceberg (Woodson et al. 2013; Leister and Kleine 2016; Grübler et al. 2017; Zhao et al. 2019; Kendrick et al. 2022). In fact, thousands of genes exhibit a substantial change in expression when chloroplast transcription/translation is disrupted, fewer than 200 of which are PhANGs. A large fraction of the genes exhibiting altered expression act outside the chloroplast, and these play diverse roles in cell biology, metabolism, and physiology. Furthermore, as many genes are upregulated as are downregulated. Among the upregulated genes are many genes involved in plastid gene expression, a phenomenon that has often been ascribed to a “compensatory” response.

We recently proposed a unifying framework that accounts for the breadth of these effects: they result from the failure to produce plastid-derived signals that are required to progress through specific “checkpoints” in the normal program of photosynthetic differentiation (Loudya et al. 2020; Kendrick et al. 2022). Thus, the altered transcriptomes of plants with disrupted chloroplast biogenesis reflect retention of juvenile states. For example, reduced PhANG expression results from the stalling of the developmental program prior to the period of peak PhANG expression. Similarly, an enhanced expression of NEP and other genes involved in plastid gene expression, rather than being compensatory, results from the stalling of the program prior to the decline in their expression (see Fig. 1).

We initially suggested this concept based on properties of the virescent Arabidopsis *cue8* mutant (Loudya et al. 2020). *cue8* is a hypomorphic allele of the gene for Tic100, which is a component of the chloroplast protein import complex (Loudya et al. 2022). The *tic100^cue8^* mutant presents a chloroplast gene expression phenotype similar to that near the base of cereal leaves: RNAs transcribed primarily by NEP are present at increased levels, while those that are primarily transcribed by PEP are reduced (Baumgartner et al. 1993; Chotewutmontri and Barkan 2016; Loudya et al. 2020). Cells in *tic100^cue8^* leaves share additional similarities to those at the base of the cereal leaf (Baumgartner et al. 1989; Loudya et al. 2021): their plastids are small, occupy a small proportion of the cellular space, and have more tightly packed nucleoids, and they exhibit an elevated expression of several nuclear genes involved in plastid protein import and a reduced expression of several PhANGs (Loudya et al. 2020). Additionally, plastid RNA editing—the posttranscriptional change of specific cytidine residues to uridine (reviewed in Small et al. 2020)—is disrupted in *tic100^cue8^*, in other plastid import mutants and when plastid translation is inhibited or norflurazon applied (Karcher and Bock 1998; Halter et al. 2004; Kakizaki et al. 2012). We speculate that the RNA editing patterns observed in such plants are characteristic of those at early stages of undisturbed plastid development, a possibility that awaits testing by characterization of RNA editing in proplastids.

**Figure 2.**
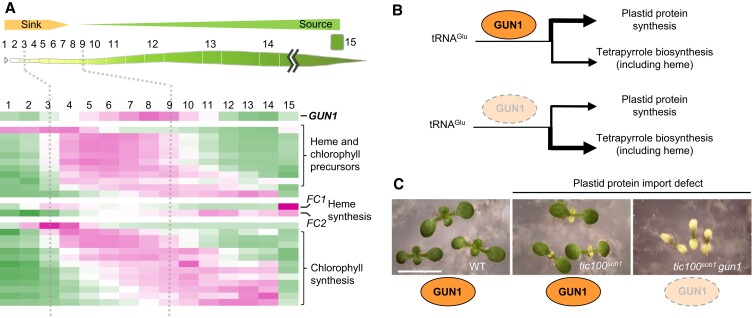
A model for GUN1's role in chloroplast biogenesis and retrograde signaling. **A)** The expression of tetrapyrrole biosynthesis genes and *GUN1* along the developmental gradient of the wheat leaf (Loudya et al. 2021). *FC1* and *FC2* are required for the synthesis of extraplastidic and intraplastidic heme, respectively (Woodson et al. 2011; Page et al. 2020; Richter et al. 2023). *FC1* peaks in expression at the stage at which development is blocked in maize mutants lacking plastid ribosomes, consistent with the view that exported heme is required to proceed past that stage. The data displayed in the heatmap are provided in Supplementary Data Set 1. **B)** A hypothetical role for GUN1 in modulating the partitioning of tRNA^Glu^ for plastid translation and tetrapyrrole synthesis. Appropriate partitioning of these functions is especially important at the time in development when *GUN1* expression peaks. **C)** GUN1 action reduces the phenotypic consequences of some chloroplast biogenesis defects. The virescent phenotype of a chloroplast protein import mutant (*tic100^soh1^*), with an early biogenesis defect that is rapidly overcome as development proceeds, is converted into a nearly albino phenotype in the *tic100^soh1^ gun1* double mutant. Images of seedlings reproduced from Loudya et al. (2022).

The concept that disruption of chloroplast biogenesis stalls the program of photosynthetic differentiation garnered strong support from our recent comparison of transcriptomes of maize chloroplast biogenesis mutants with transcriptomes along the developmental gradient of the wild-type maize leaf (Wang et al. 2014; Kendrick et al. 2022). This analysis employed 2 albino mutants with primary defects in plastid tRNA metabolism (*ppr5*) or PEP-mediated transcription (Zm-*murE*) and 2 chlorotic mutants with primary defects in thylakoid protein targeting (*tha1*) or PEP-mediated transcription (Zm-*ptac12*). All 4 mutants were nonphotosynthetic, but they represent a range of plastid gene expression defects: the albino mutants lack plastid ribosomes, Zm-*ptac12* mutants have a reduced content of plastid ribosomes, whereas *tha1* mutants have normal plastid gene expression. The mutant transcriptomes presented in 2 classes: a chlorotic syndrome shared by the 2 chlorotic mutants and an albino syndrome shared by the 2 mutants lacking plastid ribosomes. PhANG repression was observed only in the albino mutants, but genes encoding plastid-localized proteins made up only half of the genes that respond in this manner. Furthermore, many genes involved in the biogenesis of chloroplasts were upregulated in the mutants, along with many genes encoding extraplastidic proteins, including numerous genes involved in cell proliferation and cytosolic protein synthesis. Most tellingly, the transcriptomes of the albino mutants closely resembled those at very early stages of photosynthetic differentiation near the base of a wild-type leaf (Fig. 1). By contrast, transcriptomes of the chlorotic mutants implied a hybrid developmental status: expression of the Biogenesis cohort resembled that near the normal sink–source transition, whereas expression of the Photosynthesis cohort proceeded normally. *Ferrochelatase I (FC1)*, required to produce heme for export from the chloroplast (Woodson et al. 2011), peaks in expression near the base of the wheat leaf (Fig. 2), prior to peak expression of many chloroplast biogenesis genes and at the same stage at which photosynthetic differentiation stalls in maize mutants lacking plastid ribosomes (Loudya et al. 2021; Kendrick et al. 2022). This correlation is consistent with the view that heme export is the signal that allows development to proceed beyond that early stage.

Thus, this analysis revealed retention of not 1 but 2 distinct juvenile states (Fig. 1): (i) the absence of plastid translation stalls the program in the “plastid proliferation phase,” prior to the induction of PhANGs, prior to upregulation of the chloroplast assembly gene cohort, and prior to the downregulation of plastid import and division genes and (ii) the absence of photosynthesis without the loss of plastid gene expression stalls the chloroplast assembly program before the decline in expression of the Biogenesis gene cohort without altering the Photosynthesis cohort. This manifests as normal PhANG expression and elevated expression of many chloroplast assembly/gene expression functions. Thus, the loss of PhANG expression and increase in NEP expression were but the first recognized manifestations of developmental stalling, which, in fact, affect thousands of genes involved in diverse processes (Kendrick et al. 2022). These include numerous transcription factors, which presumably account for the transcriptional changes.

Finally, the analysis revealed a negative correlation between the buildup of the chloroplast gene expression system across the mutant set and the activity and transcriptional readouts of Target of Rapamycin (TOR) kinase, a central regulator of cell proliferation. In fact, the transition from cell division to cell expansion is coincident with the “chloroplast assembly” phase along the wild-type wheat leaf (Loudya et al. 2021; Fig. 1). These observations suggest that the massive consumption of amino acids and nucleotides for the production of chloroplast ribosomes and their translation products transiently represses TOR, triggering the switch from cell division to cell expansion and placing the developing chloroplast as a central regulator of this key transition (see Kendrick et al. [2022] for details). The fact that norflurazon inhibits the transition from cell division to cell expansion in Arabidopsis suggests that this theme applies also to dicots (Andriankaja et al. 2012).

This body of data, when interpreted in the context of prior findings, implies that 3 plastid-derived signals provoke distinct transitions in the nuclear transcriptome during photosynthetic differentiation (Fig. 1B). (i) The increase in PEP-mediated transcription of tRNA^Glu^ early in chloroplast development triggers the synthesis of a heme-related molecule that activates PhANGs and many other genes; expression of this gene cohort peaks in the late phase of photosynthetic differentiation in conjunction with peak chloroplast translational output. (ii) Consumption of nutrients during the buildup of the plastid translation machinery transiently inhibits TOR kinase, promoting exit from the cell proliferation phase of leaf development. (iii) A signal that reflects photosynthetic competence represses the “assembly” gene cohort (including many genes involved in plastid gene expression), shutting down the assembly program once photosynthesis is established. The nature of this photosynthesis-dependent repressive signal is not known. All told, the wide-held view that biogenic retrograde signaling serves to coordinate nuclear and plastid gene expression captures just one component of a much broader phenomenon involving multiple signals and effects on the developmental state of the entire cell.

## GUN1 through an evolutionary and developmental lens

Any discussion of biogenic retrograde signaling would be incomplete without considering the role of GUN1. GUN1 stands apart from other GUN proteins in that it is not an enzyme of the tetrapyrrole pathway (Vinti et al. 2000; Koussevitzky et al. 2007). Despite intensive study, the molecular and physiological functions of GUN1 and the mechanism by which it influences retrograde signaling remain enigmatic. As a PPR protein, the starting assumption was that GUN1 exerts its effects by binding RNA. GUN1 belongs to a PPR subfamily consisting of repeating PPR motifs followed by a Small MutS Related (SMR) domain (Koussevitzky et al. 2007). Other chloroplast PPR–SMR proteins enhance the translation, stability, or processing of specific RNAs (Zoschke et al. 2012, 2016; Wu et al. 2016), and some SMR domains have ribonuclease activity (Zhou et al. 2017; Glover et al. 2020). GUN1's PPR tract has the characteristic features of that in plant PPR proteins known to bind RNA, including the identities of amino acids at positions that specify which nucleotide is bound by each PPR motif (reviewed in Barkan and Small 2014). Thus, evolutionary considerations have long suggested that at least one aspect of GUN1 function involves its interaction with RNA.

However, negative results in attempts to detect GUN1–RNA interactions (Tadini et al. 2016) led to a shift toward an investigation of alternative interaction partners. These searches produced a plethora of putative GUN1 partners, including components of the plastid translation and import machineries (Tadini et al. 2016, 2020b; Wu et al. 2019), NEP (Tadini et al. 2020b), the RNA editing factor MORF2 (Zhao et al. 2019), enzymes involved in tetrapyrrole synthesis, and heme itself (Shimizu et al. 2019). These interactions along with phenotypes of *gun1* mutants led to a diversity of hypotheses for GUN1's function and its role in retrograde signaling. These include functions in plastid RNA editing (Zhao et al. 2019), protein import (Wu et al. 2019), NEP-mediated transcription (Tadini et al. 2020b), and reducing flux through the tetrapyrrole pathway (Terry and Smith 2013; Shimizu et al. 2019). However, as discussed by others, conflicting data and lack of critical controls weaken the case for many of these claims (Shimizu and Masuda 2021; Wu and Bock 2021; Richter et al. 2023), and a consensus has yet to emerge. Indeed, GUN1's PPR tract can be expected to engage in promiscuous interactions, as it presents a long surface that is rich in charged and polar residues. Nonphysiological interactions will be exacerbated if GUN1 is overexpressed. Thus, interaction assays that lack suitable controls (e.g. a different PPR protein of similar length) and/or that use overexpressed GUN1 as bait should be interpreted with caution.

Recent findings are pushing the pendulum back toward models invoking RNA binding as central to GUN1's activities. First, the PPR tracts of GUN1 orthologs in vascular and nonvascular plants are predicted to bind the same RNA sequence based on the amino acids at the specificity-determining positions (Honkanen and Small 2022). Furthermore, the *Marchantia polymorpha* GUN1 ortholog did not detectably influence retrograde signaling in *Marchantia* but nonetheless complemented the retrograde signaling phenotype of an Arabidopsis *gun1* mutant. The authors inferred that GUN1's ancestral function involves binding an RNA sequence that has been conserved and that the retrograde signaling effect of GUN1 is a result of the conserved RNA-binding activity (Honkanen and Small 2022). The hypothesis that GUN1 mediates its effects by binding RNA garnered additional support with the very recent discovery that Arabidopsis GUN1 affects the processing of a chloroplast *psbD* transcript; the affected processing event maps near a predicted GUN1-binding site, and recombinant GUN1 binds specifically to that RNA sequence *in vitro* (Cui et al. 2023). These results establish GUN1 as a sequence-specific RNA-binding protein. That said, GUN1's effect on *psbD* mRNA (which encodes a subunit of the PSII reaction center) is unlikely to be relevant to GUN1's effects on retrograde signaling: ribosome profiling analysis did not detect a change in the translational output of *psbD* in *gun1* mutants (relative to that of other chloroplast genes; Wu et al. 2019), and PSII biogenesis/function has not previously been linked to biogenic signaling. Given that many PPR proteins act on multiple RNAs *in vivo*, we favor the view that GUN1 has an additional as yet undiscovered RNA ligand that accounts for its retrograde signaling effects. Although the “PPR code” (Barkan et al. 2012) provides clues as to GUN1's potential RNA ligands (Honkanen and Small 2022), the current understanding of PPR–RNA interactions is not sufficient for firm prediction of physiologically relevant partners (e.g. Rojas et al. 2018).

GUN1 exhibits unusual developmental dynamics that offer additional clues as to its functions. The stability and abundance of GUN1 reporter fusion proteins change dramatically in response to the developmental status of the plastid: protein abundance and stability are at their peak in proplastids, etioplasts, and at early stages following leaf initiation, declining rapidly once chloroplast biogenesis nears completion (Wu et al. 2018; Hernández-Verdeja et al. 2022). This effect has been ascribed to regulated proteolysis as a means to provide GUN1 when it is needed. Alternatively, perhaps GUN1 is stabilized when bound to its ligand(s) such that its stability reflects its ligand concentration.

Our transcriptome analysis of highly resolved early developmental stages near the base of the wheat leaf (Loudya et al. 2021) revealed a distinctive and intriguing *GUN1* expression pattern. *GUN1* mRNA accumulates throughout the plastid proliferation phase, peaks in the assembly phase, and then rapidly declines (Fig. 1A). Although this expression pattern resembles that of many genes involved in chloroplast gene expression, it is notable for its narrower developmental window. These developmental dynamics suggest that GUN1 function is most relevant at a specific moment early in chloroplast development, shortly after the plastid gene expression system begins to ramp up. What might this moment be? It is intriguing that this is the period during which precise control of tetrapyrrole synthesis is most crucial because photosystem core subunits are just starting to be synthesized and chlorophyll precursors and unassembled chlorophylls produce damaging reactive oxygen species (ROS) upon exposure to light (Tanaka and Tanaka 2007; Hernández-Verdeja et al. 2022). Taken together with the facts that GUN1 restricts flux through the tetrapyrrole pathway, reduces the buildup of protochlorophyllide in etiolated seedlings, and enhances survival of etiolated seedlings following their exposure to light (Shimizu et al. 2019; Hernández-Verdeja et al. 2022), a hypothesis emerges for a role for GUN1 in managing tetrapyrrole metabolism at this critical juncture in chloroplast development. ROS damage from mismanaged tetrapyrrole metabolism during early leaf development might account for the curious fact that ∼10% of Arabidopsis seedlings that are homozygous for the null *gun1-101* allele are chlorotic (Ruckle et al. 2007; Wu et al. 2018).

How might these puzzle pieces fit together into a picture that joins molecular functions of GUN1 with its retrograde signaling effects? There are, no doubt, numerous solutions consistent with current data, but we suggest a possibility that incorporates the following features that we feel are supported by compelling evidence (see above): (i) a heme-related molecule is a positive signal for PhANG induction; (ii) GUN1 restricts flux through the tetrapyrrole pathway; (iii) GUN1 expression in wheat peaks at a moment in chloroplast development when precise control of tetrapyrrole synthesis is crucial; (iv) plastid tRNA^Glu^ is required for tetrapyrrole synthesis; and (v) GUN1's function involves binding RNA. As plastid tRNA^Glu^ provides a link between RNA and tetrapyrrole synthesis (including heme), it seems reasonable to propose that GUN1 affects retrograde signaling by affecting the handling of this tRNA. Management of tRNA^Glu^ activities presents a regulatory challenge as tRNA^Glu^ is required for both plastid translation and tetrapyrrole synthesis. Appropriate partitioning of these functions is critical during the initial synthesis of plastid-encoded subunits of the photosynthetic apparatus. Thus, GUN1 could affect the processing or modification of tRNA^Glu^ in a manner that influences its partitioning between these 2 functions (Fig. 2B). For example, GUN1 might bind tRNA^Glu^ or a precursor thereof to inhibit interaction with glutamyl-tRNA reductase (GluTR). In fact, such a model, in broad outline, was proposed previously (Terry and Smith 2013). That overexpression of GUN1 does not cause a chlorophyll-deficient phenotype may seem inconsistent with an inhibitory effect of GUN1 on tetrapyrrole synthesis. However, the degree to which GUN1 is functionally overexpressed in such lines may be small due to protein instability (Wu et al. 2018; Hernández-Verdeja et al. 2022). Furthermore, the high abundance of tRNA^Glu^ at later developmental stages might buffer the effects of diverting a small fraction away from tetrapyrrole synthesis. Intriguingly, a point mutation in tRNA^Glu^ that prevents processing of its termini had a dominant negative effect on tetrapyrrole synthesis when expressed from an ectopic site but did not disrupt translation (Agrawal et al. 2020). These results suggest the possibility that a GUN1-dependent precursor of tRNA^Glu^ transiently inhibits tetrapyrrole synthesis during normal chloroplast development. The chloroplast *clpP* mRNA provides another potential connection between plastid RNA and tetrapyrrole synthesis, because Clp protease influences GluTR abundance (Apitz et al. 2016). Thus, GUN1 could potentially restrict tetrapyrrole synthesis (including heme) by affecting *clpP* mRNA metabolism.

## Is GUN1's role in biogenic signaling adaptive or an emergent property of its role in tetrapyrrole metabolism?

In summary, the fundamental role of GUN1 may be to manage tetrapyrrole metabolism during a critical window early in the assembly of the photosynthetic apparatus, preventing the buildup of damaging photoactive porphyrins. This possibility aligns with the recent proposals that GUN1 acts as a “safeguard” during seedling emergence (Hernández-Verdeja et al. 2022) and that GUN1 protects chloroplasts from oxidative damage (Fortunato et al. 2022). It can also account for the fact that *gun1* exacerbates phenotypes of many chloroplast biogenesis mutants (Kakizaki et al. 2009; Loudya et al. 2020; Tadini et al. 2020b; Shimizu and Masuda 2021; Loudya et al. 2022; Richter et al. 2023; Fig. 2C). GUN1's impact on the tetrapyrrole pathway seems likely to be a result of its interaction with a chloroplast RNA (perhaps tRNA^Glu^). These activities would repress heme synthesis, thereby repressing PhANGs and coregulated genes (Fig. 2).

It is unclear whether GUN1's effects on retrograde signaling were adaptive or were an emergent property of its effects on tetrapyrrole metabolism, whose impact on the production of a biogenic signal had not been GUN1's evolutionary driving force. Although GUN1 ameliorates defects associated with various genetic impairments of chloroplast biogenesis (e.g. Fig. 2C), this is not a natural context. It is reasonable to speculate, however, that GUN1's role in retrograde signaling may be adaptive when temporary impairments occur early in development, for example in the form of nutrient deficiencies. For example, the virescence (slow-greening) observed when nitrogen is limited is at least superficially similar to the phenotypic manifestation of a range of plastid genetic defects and may trigger effects on biogenic retrograde signaling that enhance survival. It would be interesting to examine whether the action of GUN1 enhances the survival of seedlings under nutrient stress.

## Developmental stalling may underlie disparate phenotypes associated with inhibition of chloroplast biogenesis

In summary, there is evidence that 3 plastid-derived signals report distinct stages of chloroplast development to the nucleocytoplasmic compartment and that these signals usher developing photosynthetic tissues through key developmental transitions. Thus, disruption of chloroplast biogenesis results in retention of early stages of photosynthetic differentiation. Accordingly, retained juvenile states might account for effects of disrupted chloroplast biogenesis on processes such as chloroplast RNA editing (Karcher and Bock 1998; Halter et al. 2004; Kakizaki et al. 2012) and protein import (Wu et al. 2019; Tadini et al. 2020b; Loudya et al. 2022). As GUN1 inhibits one of these signals (assumed here to be heme), some effects of *gun1* on plants with disrupted chloroplast biogenesis may result from the accelerated expression of the heme-activated gene cohort, which alters the phasing of the normal stages of chloroplast development. The conceptual framework outlined here provides a unifying model that helps explain a number of hitherto disparate observations and may help guide further progress in our understanding of chloroplast biogenesis within its cellular and developmental context.

## Supplementary Material

koae094_Supplementary_Data

## Supplementary data

**Supplementary Data Set 1**. Data underlying heat maps in Figs. 1 and 2.

## Dive Curated Terms

The following phenotypic, genotypic, and functional terms are of significance to the work described in this paper:

FC1 Gramene: AT5G26030
FC1 Araport: AT5G26030

PTAC12 Gramene: AT2G34640
PTAC12 Araport: AT2G34640

THA1 Gramene: AT1G08630
THA1 Araport: AT1G08630

fc2 Gramene: AT2G30390
fc2 Araport: AT2G30390

murE Gramene: AT1G63680
murE Araport: AT1G63680

MORF2 Gramene: AT2G33430
MORF2 Araport: AT2G33430

GUN1 Gramene: AT2G31400
GUN1 Araport: AT2G31400

TIC100 Gramene: AT5G22640
TIC100 Araport: AT5G22640
